# A312 FACTORS ASSOCIATED WITH PHYSICAL SYMPTOM BURDEN AMONG PATIENTS WITH PANCREATIC CANCER

**DOI:** 10.1093/jcag/gwad061.312

**Published:** 2024-02-14

**Authors:** R Khan, M Salim, P Tanuseputro, A Hsu, R Talarico, A Gayowsky, C Webber, H Seow, R Sutradhar, P D James

**Affiliations:** University of Toronto, Toronto, ON, Canada; University of Toronto, Toronto, ON, Canada; Ottawa Hospital Research Institute, Ottawa, ON, Canada; Ottawa Hospital Research Institute, Ottawa, ON, Canada; Institute for Clinical Evaluative Sciences, Hamilton, ON, Canada; Institute for Clinical Evaluative Sciences, Hamilton, ON, Canada; Ottawa Hospital Research Institute, Ottawa, ON, Canada; McMaster University Department of Oncology, Hamilton, ON, Canada; Institute for Health Policy, Evaluation and Management, University of Toronto, Toronto, ON, Canada; University of Toronto, Toronto, ON, Canada

## Abstract

**Background:**

Patients with pancreatic ductal adenocarcinoma (PDAC) experience substantial physical symptoms. Factors associated with symptom burden in PDAC are not well described.

**Aims:**

To evaluate risk factors for symptoms assessed through the Edmonton Symptom Assessment Scales (ESAS) among patients with PDAC.

**Methods:**

We included patients diagnosed with PDAC and associated ESAS forms at diagnosis between January 1 2007 to December 31 2020 in Ontario, Canada. The ESAS contains nine symptoms (pain, tiredness, nausea, depression, anxiety, drowsiness, loss of appetite, well being, and shortness of breath) on a scale from 0 to 10. Our outcomes were moderate to severe (ESAS scores 4 and above) physical symptoms (pain, tiredness, nausea, poor appetite, drowsiness, and dyspnea). We analyzed associations between baseline variables and outcomes using multivariable regression models. Covariates included age, sex, tumour location, cancer stage, index cancer-directed therapy (surgery, surgery and chemotherapy, chemotherapy alone, radiation, and no cancer-directed therapy), distance to cancer centre, comorbidities, and access to a family physician.

**Results:**

We identified 4918 patients (mean age 68 years, 52% male) with one or more ESAS records. Most patients (61%) had stage III or IV disease at diagnosis. At diagnosis, 30%, 37%, 14%, 34%, 25%, and 15% reported moderate to severe pain, tiredness, nausea, poor appetite, drowsiness, and shortness of breath respectively. Six months after diagnosis, 42%, 59%, 25%, 51%, 41%, and 23% reported moderate to severe pain, tiredness, nausea, poor appetite, drowsiness, and dyspnea respectively. At 6 months, moderate to severe pain was associated with baseline dyspnea (p=0.003) and pain (pampersand:003C0.001), moderate to severe tiredness was associated with increasing age (p=0.04), female sex (p=0.02), pre-existing coronary disease (p=0.030), and baseline pain (p=0.010), and tiredness (pampersand:003C0.001), moderate to severe nausea was associated with female sex (pampersand:003C0.001), pre-existing osteoarthritis (p=0.010), and baseline depression (0.006), nausea (pampersand:003C0.001), and pain (pampersand:003C0.001), moderate to severe poor appetite was associated with female sex (pampersand:003C0.001), and baseline poor appetite (pampersand:003C0.001) and tiredness (p=0.490), moderate to severe drowsiness was associated with receipt of radiation therapy (p=0.003), pre-existing arrhythmia (p=0.026), and baseline depression (p=0.029), drowsiness (pampersand:003C0.001), and dyspnea (p=0.010), and moderate to severe dyspnea was associated with age (p=0.010), pre-existing COPD (pampersand:003C0.001), and baseline dyspnea (pampersand:003C0.001), and tiredness (p=0.023).

**Conclusions:**

Patients with PDAC and physical symptoms at diagnosis are more likely to have corresponding moderate to severe symptoms later on. Universal physician symptom screening may enable improved symptom management.

**Funding Agencies:**

CIHR

